# Resurfacing promotes antibacterial activity of a lipid A–binding nanobody

**DOI:** 10.1073/pnas.2509305122

**Published:** 2025-09-03

**Authors:** Angela C. O’Donnell, Xun Wang, Nikol Kadeřábková, Kyra E. Groover, Bethany C. Perez, Amanda Helms, Jennifer S. Brodbelt, Despoina A. I. Mavridou, Bryan W. Davies

**Affiliations:** ^a^Department of Molecular Biosciences, The University of Texas at Austin, Austin, TX 78712; ^b^Department of Chemistry, The University of Texas at Austin, Austin, TX 78712; ^c^John Ring LaMontagne Center for Infectious Diseases, The University of Texas at Austin, Austin, TX 78712

**Keywords:** nanobody, resurfacing, lipid A, membrane disruption, antibacterial

## Abstract

Nanobodies have been pursued as candidates for antimicrobial design due to their small size and versatile binding capacities, but direct antibacterial activity of a nanobody has yet to be described. Here, we employed a bacterial surface display platform to screen a synthetic library of nanobody variants for antimicrobial potential. We identified a candidate that binds the essential lipid A component of gram-negative lipopolysaccharide. Nonetheless, this nanobody required a weakened outer membrane to access its target and elicit its toxic activity. Borrowing from observations of innate immune proteins, we found that resurfacing nanobodies with positively charged residues enabled them to bind and perturb the gram-negative outer membrane, but this alone was not sufficient for toxic activity. However, when we resurface our lipid A-targeting nanobody, it gained the ability to disrupt the outer membrane and enact its antibacterial function against wild-type bacteria. This development of a dual-function nanobody that can reach and bind previously inaccessible gram-negative targets introduces a route for antimicrobial biologic advancement.

## Significance

Antibodies have been explored to treat antibiotic-resistant infections, but identifying leads with direct antibacterial action has proven challenging. Here, we screened a library of nanobodies for antimicrobial potential and identified a nanobody that binds lipid A, a conserved moiety at the core of the outer membrane. This nanobody was bactericidal against cells with weakened membranes, but not against intact cells. To overcome this barrier, we applied natural immune chemistry and resurfaced the nanobody scaffold with cationic charge. This enabled the nanobody to access lipid A and directly kill wild-type bacteria. Our work introduces a path toward antimicrobial antibody design, unveiling access to targets typically concealed by the outer membrane.

The rise in antimicrobial resistance and stagnation of new antibiotic development have led to the investigation of nontraditional modalities, such as peptides, phages, and antibodies, to aid in the fight against bacterial infections. Antibodies are a key component of host immune response to infection and have been leveraged to treat a wide range of diseases. Several antibodies have been developed to bind bacterial toxins to mitigate their cytotoxic effects, while others have been developed to inhibit bacterial virulence factors and/or promote immune system clearance (1–4). However, the development of antibodies that can target essential bacterial processes and directly kill has been elusive (5–7).

Two excellent studies described the investigations of antibodies that bind essential gram-negative outer membrane proteins BamA and LptD (5, 6). The BamA study successfully identified an antibody that bound BamA and inhibited *Escherichia coli* growth, but this effect was only observed in strains displaying truncated lipopolysaccharide (LPS) and altered membrane fluidity (6). This suggested that the target epitope was normally hidden below the surface of an intact bacterial outer membrane. The LptD study similarly concluded that other essential epitopes that could be targeted by antibodies were likewise hidden below the membrane surface (5). This physiology may have evolved to help bacteria evade the immune system and has thus far frustrated the development of antibacterial antibodies.

Our immune system has evolved means to overcome the gram-negative outer membrane using cationic charge (8, 9). Innate immune defense proteins, including antibacterial peptide LL37 (10, 11) and bactericidal permeability-increasing protein (BPI) (12–14), encode high numbers of cationic residues. This charge promotes their binding and penetration of the negatively charged bacterial outer membrane to reach and disrupt the inner membrane, which ultimately causes cell death. The unique properties conferred by positive charge have inspired attempts to resurface other proteins with cationic residues to endow them with membrane-permeating characteristics (15). Nonetheless, generating stable cationic resurfaced proteins is challenging, and to date, only two examples are well described: green fluorescent protein (GFP) (16) and nanobodies (17). Nanobodies are single-chain antibody fragments, which, like full-length antibodies, can be selected to bind specific targets (18, 19). We reasoned that we could identify nanobodies that bind to essential factors buried below the gram-negative outer membrane and resurface them with positively charged residues to provide a means for their action. Here, we first demonstrate that nanobodies that bind essential bacterial factors and cause cell death can be identified from large mutational libraries. Resurfacing these nanobodies with cationic charge allows them to permeate the outer membrane and reach these essential features in wild-type *E. coli*. This work demonstrates how natural immune processes can be leveraged to enhance approaches for antibody selection to develop strategies for killing bacteria.

## Results

### Antimicrobial Nanobody Candidates Can be Identified Using Surface Display.

**Fig. 1.**
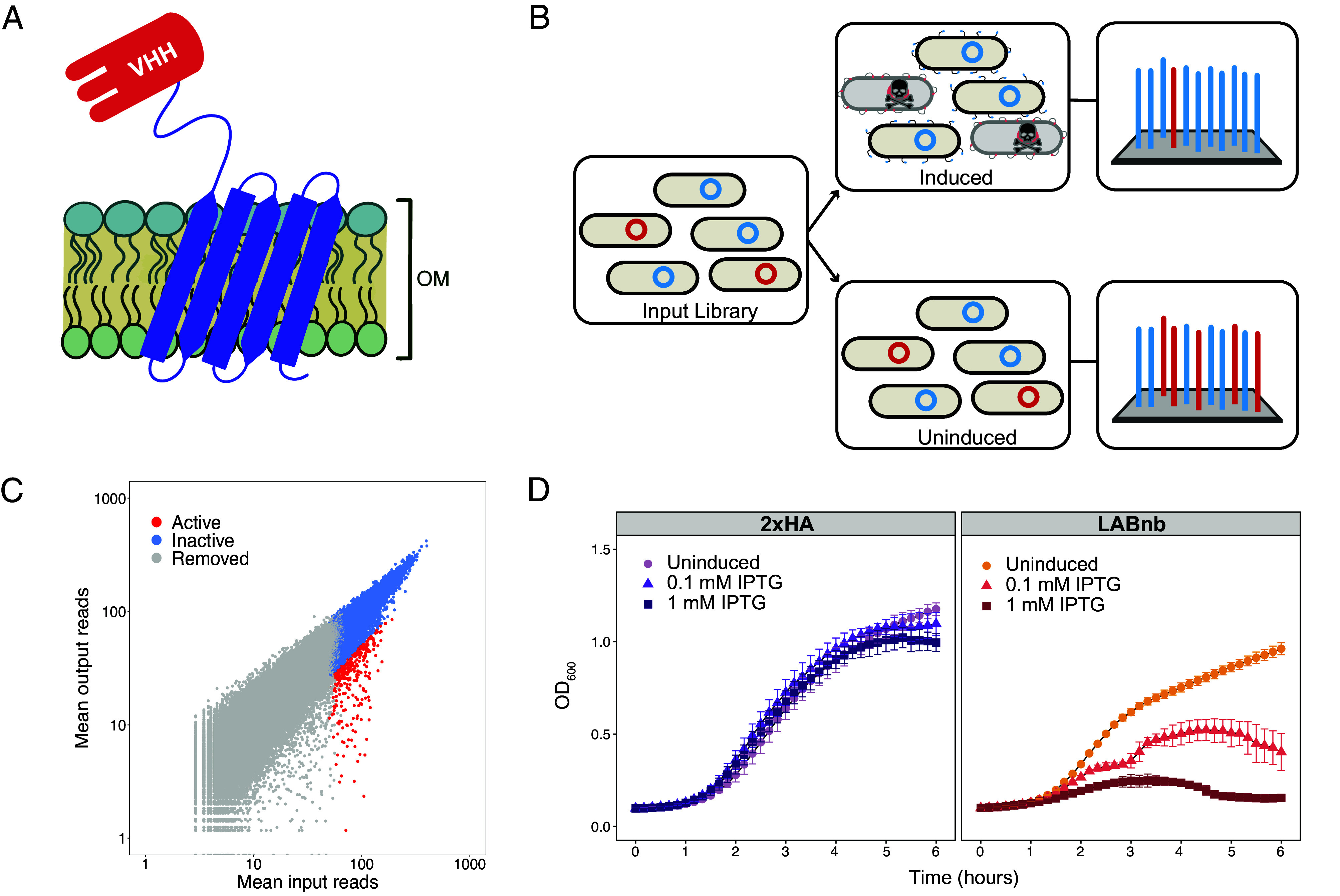
(*A*) Cartoon depicting a nanobody (VHH) tethered to the outer membrane using the SLAY display system. (*B*) Schematic of the SLAY screen. Antimicrobial leads are identified by comparing sequencing data from an uninduced input population to an induced population where antimicrobial proteins reduce the abundance of the producing cell. (*C*) Results of our nanobody SLAY screen showing mean input reads and mean output reads. Constructs with fewer than 50 reads were eliminated from analysis (gray). Log2 fold change was used to identify inactive variants (blue) and potentially antimicrobial candidates (red). (*D*) Growth curves of *E. coli* W3110 with or without induction of the SLAY system. Induction of LABnb display leads to a dose-dependent reduction in bacterial growth. Conversely, induced display of 2xHA (a tandem influenza hemagglutinin peptide) does not impact *E. coli* growth.

We previously developed Surface Localized Antimicrobial Display (SLAY), a cell-based screen to identify antibacterial peptides from large libraries (20, 21). Our approach displays peptides on the surfaces of *E. coli* cells by tethering them to a fragment of outer membrane protein A (OmpA; Fig. 1*A*) (22). This configuration allows displayed peptides to interact with the bacterial outer membrane and, if the displayed peptide inhibits bacterial growth, the bacterium expressing it is depleted from the culture (Fig. 1*B*). Previous work showed that a similar protein anchoring system could be used to display functional nanobodies that could bind their respective antigens (23). We repeated this approach and found that a nanobody recognizing GFP (24) (GFPnb) retains its ability to bind GFP when displayed from our platform (*SI Appendix*, Fig. S1). Building from this foundation, we hypothesized we could apply our antimicrobial surface display approach to recover antibacterial nanobody leads.

To explore this application, we built a synthetic nanobody library based on a previously described scaffold (18), which we have used to identify nanobodies capable of binding specific bacteria (25). In this scaffold, complementarity-determining regions (CDRs) 1 and 2 each encode 7 amino acids and CDR 3 encodes 9 amino acids (*SI Appendix*, Fig. S2*A*). We based the amino acid content of the CDRs in our library on the sequence composition profiles observed for natural nanobody constructs. Our display library consisted of ~100,000 nanobody variants displayed on *E. coli* W3110; this strain has previously been used in productive surface display screens (20). We grew replicate cultures with or without isopropyl β-D-1-thiogalactopyranoside (IPTG) induction, then recovered plasmids from each culture and sequenced the encoded nanobody CDRs. Nucleic acid sequences were translated, and nanobody variants (from CDRs 1, 2, and 3) were counted and compared between induced and uninduced conditions to identify nanobodies depleted during the assay (Fig. 1 *B* and 1 *C*).

Differences in nanobody reads between treatment conditions were evaluated by analyzing log2 fold change values. Variants that were observed to be depleted ≥ twofold in the induced population were considered as prospective antimicrobial constructs. From this collection, we selected ten nanobody sequences to test for purification. Our previous work with surface display screens showed that log2 fold change values do not always align with the activity of the purified antibacterial (20, 21). Therefore, we prioritized testing nanobodies with diverse CDR sequences over a range of log2 fold change depletions. All putative active sequences identified in our screen and the variants selected for purification are shown in Dataset S1. Only one of these constructs expressed sufficiently for purification (*SI Appendix*, Fig. S3 *A* and S3 *B*). This candidate was called lipid A–binding nanobody (LABnb) and its sequence is shown in *SI Appendix*, Fig. S2*B*. Consistent with its classification as an inhibitory protein, LABnb exhibits antibacterial activity against *E. coli* W3110 when the nanobody is displayed on the cell surface (Fig. 1*D*). This effect is visible in the uninduced culture, likely due to the documented leaky expression of plasmid pMMB67EH (26), and is amplified in the IPTG-induced cultures.

### LABnb Requires Outer Membrane Disruption for Antibacterial Action.

**Fig. 2.**
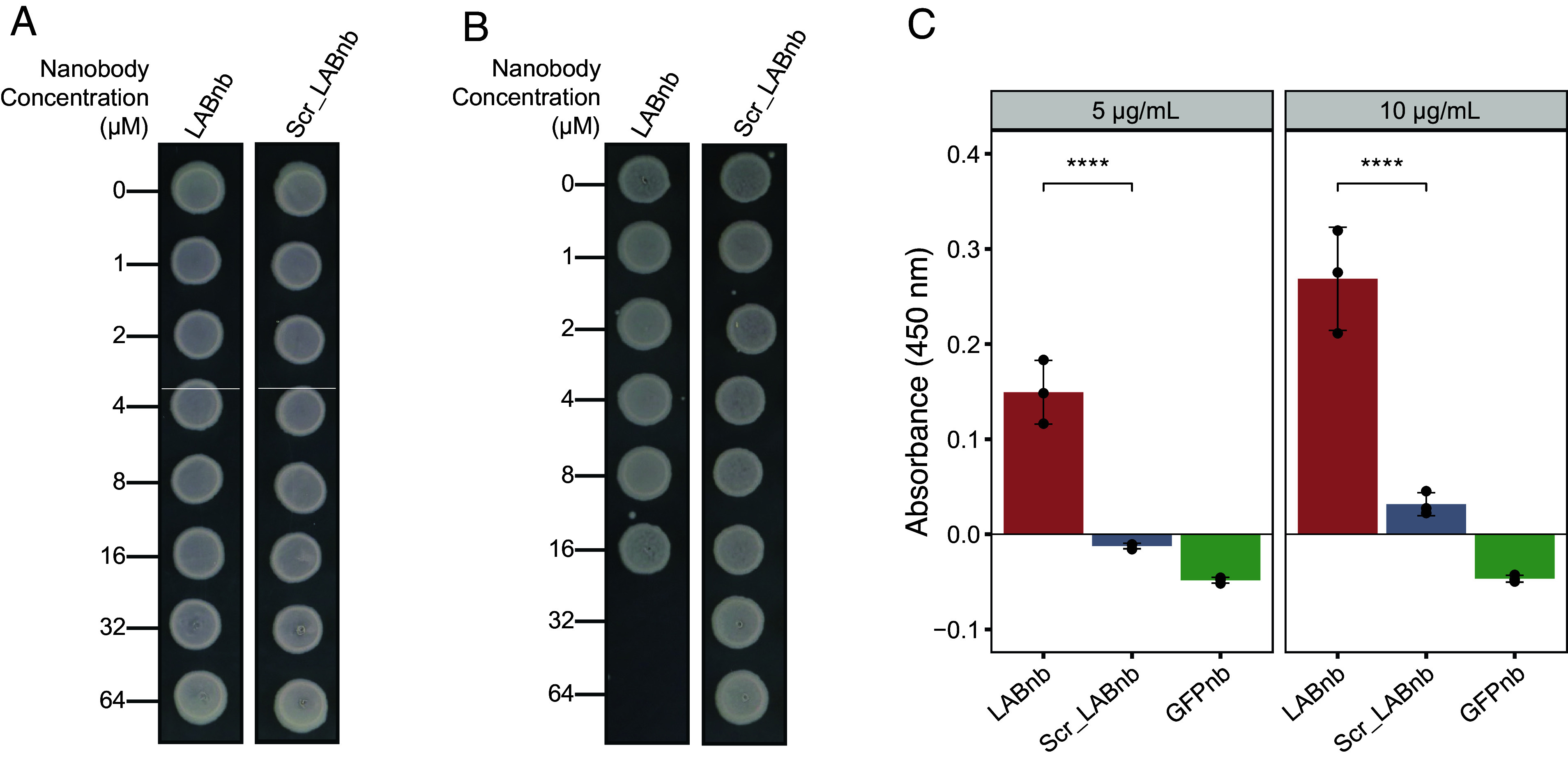
(*A*) MBC results showing that treatment with purified LABnb or scrambled LABnb (Scr_LABnb) does not affect *E. coli* survival when tested in Mueller Hinton growth medium up to 64 µM (~909 µg/mL) nanobody concentrations. (*B*) MBC assay results showing treatment with purified LABnb kills *E. coli* W3110 when the bacteria are sensitized with Tris buffer. Scrambled LABnb is not bactericidal under these conditions. (*C*) ELISA results showing LAB nanobodies binding to lipid A at two nanobody concentrations. Background absorbance was subtracted from reads. Error bars denote the SD, and individual points represent replicate data within an experiment. LABnb binds lipid A, while scrambled LABnb nanobody binds significantly less, and the anti-GFP nanobody shows no binding. Significance was determined using a two-way ANOVA with Tukey post hoc test (****P ≤ 0.0001).

While LABnb inhibited *E. coli* W3110 when displayed on the cell surface, the purified nanobody had no inhibitory effect when tested by standard minimum inhibitory concentration (MIC) and minimum bactericidal concentration (MBC) assays (Fig. 2*A*). Because the OmpA display module used in our system can cause outer membrane weakness (22), we reasoned that LABnb likely requires a perturbed outer membrane to reach its target and elicit antibacterial activity, in a fashion similar to the recently discovered BamA antibodies (6). To test this hypothesis, we repeated our MBC assay in Tris buffer, which has been shown to destabilize the outer membrane (27, 28). Under these conditions, LABnb killed *E. coli* (Fig. 2*B*), implying that the antibacterial target of this nanobody likely resides below the surface of the outer membrane. By contrast, a purified LABnb variant encoding scrambled CDR sequences (*SI Appendix*, Fig. S4*A*) did not show antibacterial activity (Fig. 2*B*), suggesting that the specificity of LABnb CDR sequences is required for its activity.

### LABnb Binds Lipid A.

We hypothesized that LABnb exerts its antibacterial action by targeting essential components of cell envelope proteins typically concealed by the outer membrane. Nonetheless, we were unable to identify the interaction partner of LABnb via immunoprecipitation (*SI Appendix*, Fig. S5), thus excluding a proteinaceous target. In addition to proteins, the cell envelope of *E. coli* contains lipids, including the LPS molecules that comprise the outer leaflet of the outer membrane. We considered that LABnb might target lipid A, an essential moiety of LPS embedded in the outer membrane, and the target of the antibiotic colistin (29, 30). An enzyme-linked immunosorbent assay (ELISA) demonstrated that, indeed, LABnb specifically bound purified lipid A (Fig. 2*C*). This binding capacity was lost in the scrambled LABnb variant and absent in the GFPnb, which naturally has a different target, suggesting that the inhibitory activity observed for LABnb may be driven by specific recognition of lipid A in the outer membrane (Fig. 2*C*). Because the CDR sequences of LABnb lack positive charge (*SI Appendix*, Fig. S2*B*), we posit that its inhibitory activity is different than the electrostatic mechanism driving the bactericidal activity of other lipid A-targeting antibacterials like colistin (29, 30).

### Resurfaced Nanobodies Disrupt the *E. coli* Outer Membrane.

Our results indicate that LABnb is only antibacterial against *E. coli* when the outer membrane is weakened (Fig. 2*B*), i.e., when lipid A is more accessible. Our immune system uses cationic charge to enable peptides and proteins to penetrate and disrupt bacterial membranes (9, 13, 31). We questioned whether conferring cationic charge to nanobodies would provide the same effect and enable LABnb to access lipid A at the core of the outer leaflet. Although most proteins are not amenable to cationic resurfacing, nanobodies have been shown to tolerate these modifications while maintaining their function (17).

To independently investigate the effects of cationic resurfacing, we designed charged variants of two benign, nonantibacterial nanobodies: the anti-GFP nanobody described above and Ng2 nanobody (Ng2nb) (25), a nanobody from our lab that encodes the same scaffold as LABnb but lacks any antibacterial activity (18). We resurfaced the scaffold of GFPnb as previously described (17) and modified the Ng2nb scaffold at comparable positions (*SI Appendix*, Fig. S6 *A*–*C*). Resurfacing shifted the theoretical net charges of GFPnb to +12.52 (from +1.52) and Ng2nb to +9.51 (from −0.49). All nanobodies migrated near their anticipated molecular weights, as determined by SDS-PAGE (*SI Appendix*, Fig. S7*A*), and were further confirmed by immunoblotting (*SI Appendix*, Fig. S7*B*). Notably, the resurfaced nanobody variants migrated slightly slower, which is likely an effect of their increased cationic charge. Purified resurfaced and nonresurfaced variants showed the anticipated β-sheet structure for nanobodies, as demonstrated by circular dichroism spectra, suggesting that resurfacing did not greatly disrupt nanobody folding (*SI Appendix*, Fig. S7*C*).

**Fig. 3.**
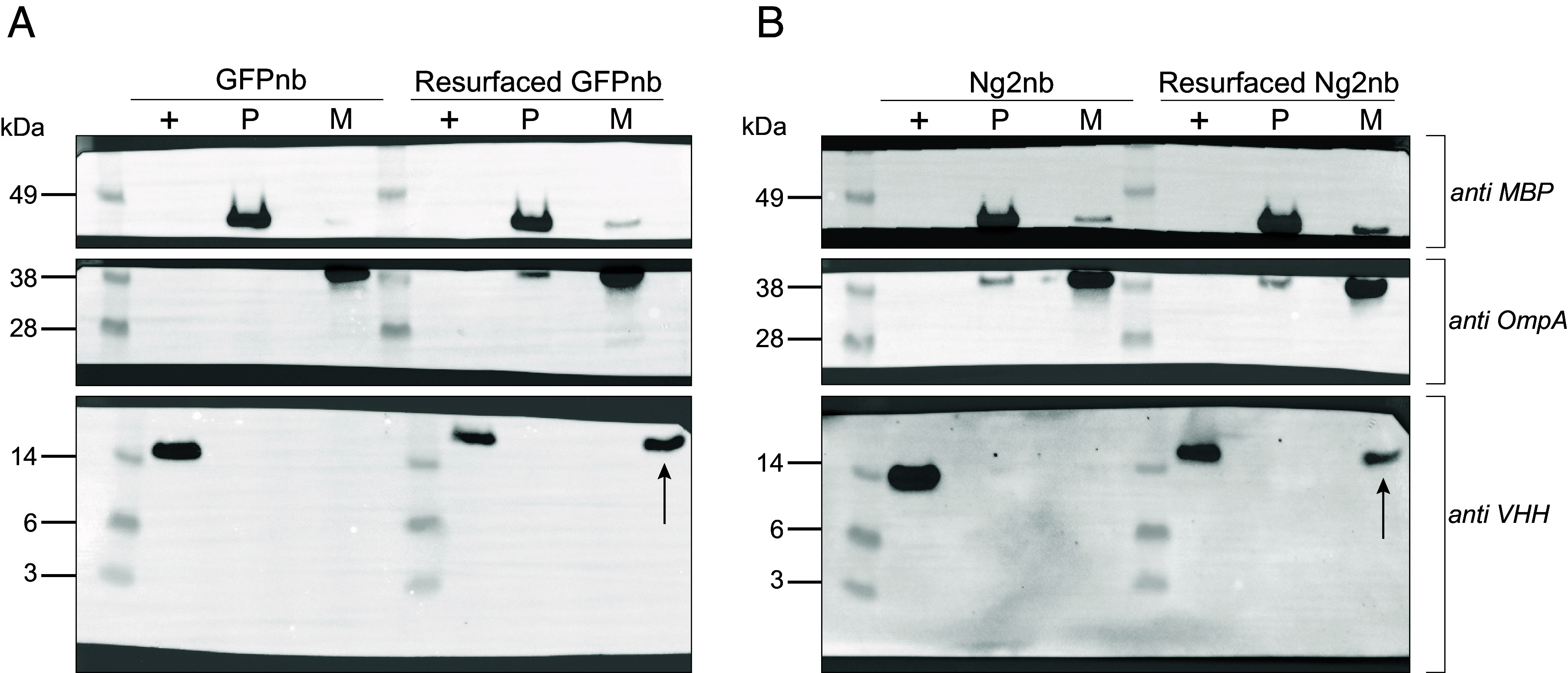
Immunoblot analysis of fractionation samples. Purified nanobodies (+) were used as a control for nanobody detection. Periplasm fractions (P) and total membrane fractions (M) were taken after treating *E. coli* W3110 with equal concentrations of nanobodies. Transfer membranes were divided and incubated with antibodies recognizing either maltose-binding protein (MBP), OmpA, or nanobodies (VHH). (*A*) Fractionation data for GFPnb and resurfaced GFPnb nanobody treatments show that resurfaced proteins are found in the membrane fraction. (*B*) Treatments with Ng2 and resurfaced Ng2 nanobodies show that resurfaced proteins are again detected in the membrane fraction. The arrows point to resurfaced nanobodies detected in total membrane components.

We first investigated whether resurfacing alone altered nanobody-*E. coli* interactions. We treated *E. coli* W3110 cells with equal molar concentrations of each nanobody and fractionated the cells to isolate periplasmic and total membrane fractions. Cellular fractions were, then, immunoblotted to determine nanobody localization. Our analyses showed that resurfaced, but not parent nanobodies, were readily observed in membrane fractions (Fig. 3 *A* and *B*), but neither were observed in periplasmic fractions. Together, these observations supported the idea that cationic resurfacing promotes nanobody interactions with anionic bacterial membranes.

**Fig. 4.**
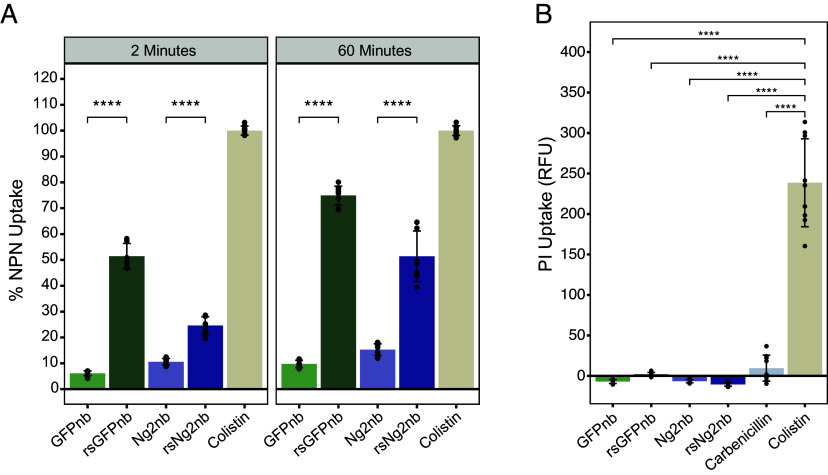
(*A*) NPN fluorescence observed for *E. coli* W3110 cells treated with purified nanobodies. Percent NPN uptake is calculated as observed fluorescence/fluorescence of colistin x 100 (background subtracted). NPN fluorescence is observed when cells are treated with resurfaced nanobodies (rsGFPnb or rsNg2nb). Error bars denote SD, and points on graph represent individual reads, with three separate experiments in triplicate displayed on the graph. (*B*) PI fluorescence represented as relative fluorescence units (RFU). All nanobodies and the carbenicillin control display negligible fluorescence, while strong signal is observed for colistin, a known inner membrane disruptor. Background fluorescence generated by untreated cells was subtracted from reads. Points represent individual reads from three experiments conducted in triplicate. Significance was determined using a two-way ANOVA (for NPN) or one-way ANOVA (for PI), with Tukey post hoc tests (****P ≤ 0.0001).

Cationic proteins can bind and disrupt bacterial membranes, but our fractionation assays did not measure this property or deconvolute whether these interactions were localized to the inner or outer membranes. To provide further resolution for resurfaced nanobody interactions within the cell envelope, we first tested for outer membrane disruption using 1-*N*-phenylnaphthylamine (NPN), a nonpolar probe that fluoresces within phospholipid environments (32). In the presence of an intact outer membrane, weak NPN signal is detected, but, if the outer leaflet is disrupted such that the phospholipids within the inner leaflet become exposed, strong NPN fluorescence is observed (32). We compared fluorescence against a high concentration of colistin, a potent membrane disruptor, which served as the estimated maximum NPN signal expected for complete membrane disruption. While *E. coli* cells treated with NPN and nonresurfaced nanobodies generated negligible fluorescent signal, treatment with resurfaced GFPnb or resurfaced Ng2nb resulted in strong fluorescence (Fig. 4*A*), supporting a model in which resurfaced nanobodies can disrupt the bacterial outer membrane.

We next considered the possibility that resurfaced nanobodies also disrupt bacterial inner membranes. To test this, we used propidium iodide (PI), a membrane-impermeant molecule that fluoresces when allowed to intercalate DNA. Inner membrane disruption induced by a colistin treatment resulted in strong fluorescent signal from PI (Fig. 4*B*). Conversely, *E. coli* treated with purified nanobodies did not appear to generate a PI fluorescent signal, regardless of scaffold resurfacing (Fig. 4*B*). Collectively, our data show that cationic resurfacing grants nanobodies the ability to interact with gram-negative bacteria at the outer membrane but does not result in periplasm entry or inner membrane disruption. We provide a comparison of colistin and polymyxin B nonapeptide for these assays in *SI Appendix*, Fig. S8 *A* and *B*.

Scaffold resurfacing allowed nanobodies to interact with and disrupt outer membranes; however, no MIC or MBC was detected, up to 64 µM nanobody concentrations, for *E. coli* W3110 treated with resurfaced or nonresurfaced GFPnb or Ng2nb proteins (*SI Appendix*, Fig. S9). To further investigate the extent of membrane perturbation, we tested whether resurfaced nanobodies could potentiate the activity of vancomycin, an antibiotic that cannot intrinsically cross the outer membrane. However, we did not detect bactericidal activity (*SI Appendix*, Fig. S10), suggesting that the observed membrane disruption does not allow all molecules across the entire outer membrane.

### Resurfaced LABnb Exhibits Antimicrobial Activity.

Considering that membrane disruption was required for LABnb to elicit antibacterial activity and that resurfaced nanobodies gain the ability to perturb *E. coli* outer membranes, we hypothesized that resurfacing LABnb would enable its activity against intact bacteria. Using the resurfacing designs described above, we substituted residues within the LABnb scaffold for positively charged amino acids, thereby shifting the theoretical net charge of the protein from −3.57 to +6.42 (*SI Appendix*, Fig. S4 *A* and *B*). We resurfaced the scrambled LABnb variant using the same design scheme to serve as a control for antimicrobial activity assays (*SI Appendix*, Fig. S4*A*). Although resurfacing led to significant decreases in LABnb purification yields (~fourfold), we still obtained sufficient quantities for antibacterial testing against the original screening strain, *E. coli* W3110.

**Fig. 5.**
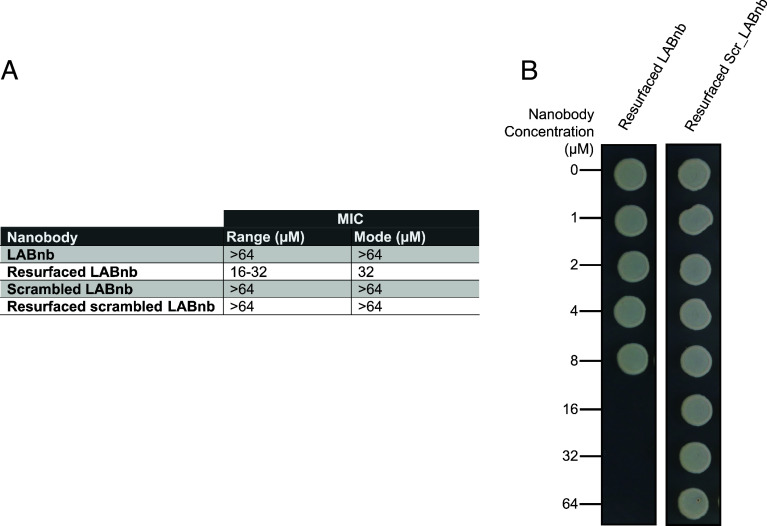
(*A*) Observed MIC values for each nanobody against *E. coli* W3110. No MIC was detected for the nonresurfaced LABnb variant or either of the scrambled constructs. MIC values ranged between 16 and 32 µM (234 to 468 µg/mL) for resurfaced LABnb, with 32 µM representing the most common MIC. (*B*) Representative MBC results for *E. coli* W3110 treated with purified resurfaced LABnb and its scrambled variant. Resurfaced LABnb displays bactericidal activity at 16 µM concentration. The scrambled variant does not display bactericidal activity at the tested concentrations (>64 µM, or 935 µg/mL).

Using standard MIC and MBC assays, we found that resurfaced LABnb elicited antimicrobial activity against *E. coli*, meanwhile its scrambled counterpart did not. Although MIC values varied ~twofold between separate purification efforts, an MIC range of 16 to 32 µM (i.e., 234 −468 µg/mL) was observed for *E. coli* W3110 (Fig. 5*A*). MBC activity for resurfaced LABnb was generally twofold higher than recorded MIC values and ranged from 16 to 64 µM (i.e., 234 to 935 µg/mL) across separate purifications (Fig. 5*B*). Antimicrobial activity was specific to resurfaced LABnb and no inhibitory MIC activity (>64 µM, or 935 µg/mL) was observed for any of the scrambled LABnb variants (Fig. 5 *A* and *B*).

To explore whether this antibacterial activity could be applied to other gram-negative species, we conducted antibacterial assays using two additional gram-negative strains: *Acinetobacter baumannii* and *Pseudomonas aeruginosa*. We found that MIC and MBC activity of resurfaced LABnb against *A. baumannii* was similar to that observed for *E. coli*, but the MIC for *P. aeruginosa* was ~twofold higher and no MBC was detected (*SI Appendix*, Fig. S11 *A*–*C*). Similarities in lipid A structure between these strains may indicate that the observed differences in resurfaced LABnb sensitivity are not due to target recognition (33–35). We extended this analysis to two gram-positive strains, which inherently lack outer membranes and LPS. Both parental LABnb and scrambled LABnb resurfaced nanobodies demonstrated inhibitory activity against *Staphylococcus aureus* and *Staphylococcus epidermidis*, with more potent activity observed for the resurfaced scrambled LABnb variant (*SI Appendix*, Fig. S11*A*). However, no MBC was observed for either nanobody against either *Staphylococcus* species (*SI Appendix*, Fig. S11 *D* and *E*). This suggests a general static action of resurfaced nanobodies against these gram-positive strains, but no targeted antibacterial action. Cationic peptides frequently show general membrane-disrupting activity and off-target toxicity (36, 37). To begin to test whether the membrane-disrupting effects of resurfaced nanobodies are limited to bacterial cells, we tested their action against red blood cells. At our maximum tested nanobody concertation of 64 µM, neither resurfaced LABnb nor its scrambled counterpart disrupted human red blood cells (*SI Appendix*, Fig. S12), indicating that nanobodies do not generally disrupt mammalian cells.

**Fig. 6.**
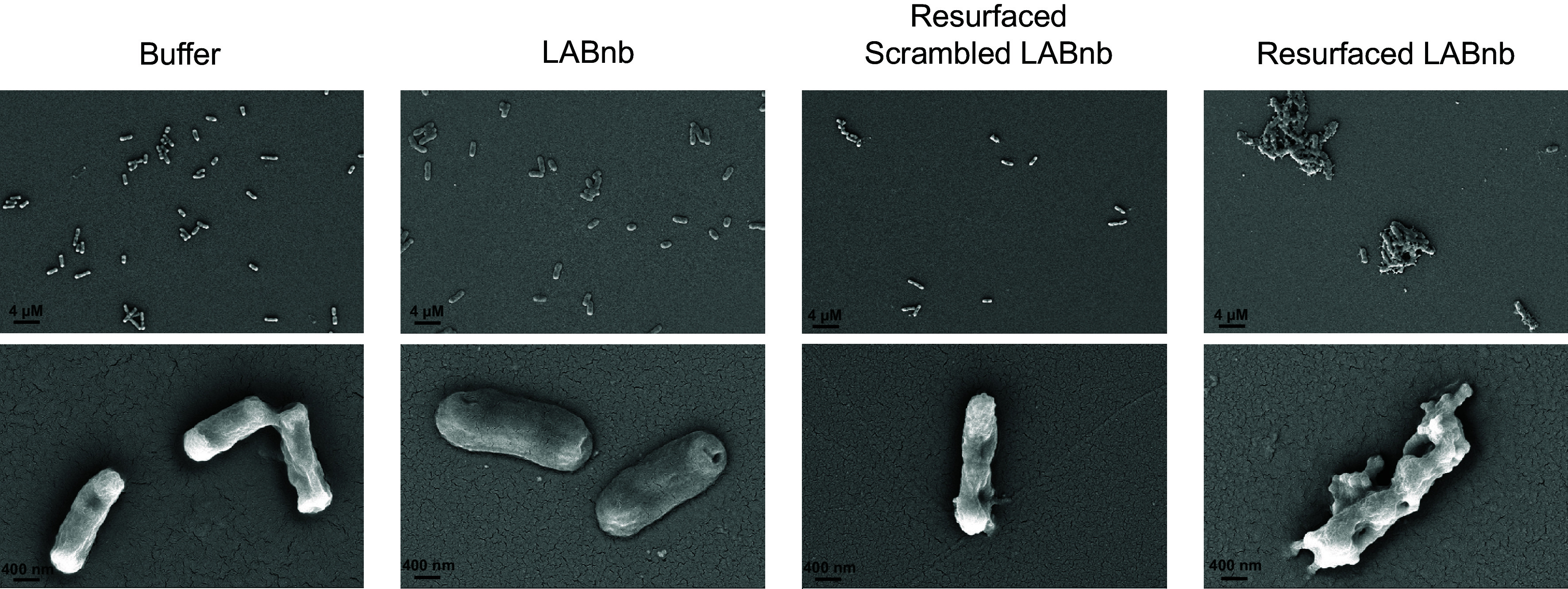
SEM images displaying the effects of nanobody treatments on *E. coli* W3110. Untreated cells (buffer) are shown in the leftmost panel. Treatments with LABnb show minimal perturbation of the cell surface. Resurfaced scrambled LABnb nanobodies induce minor membrane disruption when compared to resurfaced LABnb nanobodies, which clearly display membrane damage distinct from other treatments.

Finally, to visualize the effects of resurfaced LABnb, we exposed *E. coli* W3110 to purified nanobodies and imaged the treated cells using scanning electron microscopy (SEM). The images clearly show that resurfaced LABnb induces membrane blebbing and cell content leaking consistent with significant membrane damage (Fig. 6). By comparison, cells treated with buffer alone or nonresurfaced LABnb possess intact membranes (Fig. 6). The effects of nanobody scaffold resurfacing on *E. coli* are captured in treatments with either resurfaced scrambled LABnb (Fig. 6) or resurfaced GFPnb (*SI Appendix*, Fig. S13), where slight distortion of the cell membranes is visible. This is supported by our NPN and fractionation data, which show that resurfacing alone contributes to some degree of membrane perturbation. Nonetheless, these effects are evidently distinct from the resurfaced LABnb-treated cells, which show intense clumping and severely disrupted cell membranes (Fig. 6). Overall, our data show that scaffold resurfacing and lipid A binding specificity together confer the ability for nanobodies to exert antimicrobial activity against gram-negative bacteria. Moreover, we also demonstrate the effects of colistin on *E. coli,* which, as anticipated, results in membrane disruption (*SI Appendix*, Fig. S13). However, these effects appear distinct in morphology from those induced by resurfaced LABnb. This phenotypic difference reinforces a membrane disruption model distinct from the killing mechanism of colistin.

Together, our data support a cell-killing model in which nanobody scaffold resurfacing and CDR recognition of lipid A together compound the effects of membrane disruption, ultimately resulting in cell death. We therefore tested whether antimicrobial activity could be achieved by treating Tris-sensitized *E. coli* with nonspecific resurfaced nanobodies. Both resurfaced GFPnb and resurfaced scrambled LABnb exerted antibacterial activity against sensitized cells (*SI Appendix*, Fig. S14), indicating that additional membrane disruption is needed for resurfaced nanobodies to be antibacterial. This suggests that membrane-disrupting effects of lipid A binding by LABnb in combination with scaffold resurfacing confer an additive effect that destabilizes the membrane sufficiently to kill the bacterial cell.

## Discussion

Antibodies have been explored as treatment strategies to address antimicrobial resistance; however, most examples are limited to toxin neutralization (38–40) and colonization prevention (1, 3, 41). This remains true for the three FDA-approved antibacterial antibodies, which bind toxins produced by *Bacillus anthracis* [i.e., raxibacumab (2) and obiltoxaximab (42)] or *Clostridioides difficile* [i.e., bezlotoxumab (43)]. Of the few directly antibacterial antibody examples that have been presented, limitations in their activities, including inaccessibility of binding targets (5, 6), have hampered their pursuit as antimicrobial therapies. More recently, single-chain variable fragments (scFvs) that bind *A. baumannii* were shown to inhibit bacterial growth in wild-type strains (44). This activity was attributed to antibody cationic charge, but no MIC or identification of CDR-recognized targets were reported.

In this work, we identified a lipid A–binding nanobody from a large synthetic library and resurfaced its scaffold with cationic residues. While cationic resurfacing conferred the ability for nanobodies to permeate the gram-negative outer membrane, only the resurfaced lipid A–binding nanobody elicited bactericidal activity. Because interactions between a bacterium and its environment are defined by the outer membrane, overcoming this nearly impermeable structure is one of the most prominent barriers toward therapeutic development (45, 46). This impermeability results from LPS molecules that decorate the bacterial surface and are further linked by divalent cations to form strong lateral interactions that strictly limit access to the cell interior (47–49). Positively charged molecules, such as cationic antimicrobial peptides, can displace these interactions and destabilize the integrity of the outer membrane (49). It is therefore reasonable to propose that the positively charged nanobody scaffold allows the protein to, to some degree, embed the outer membrane, but an additional mode of disruption is required for bactericidal activity. This is supported by our fractionation and MIC/MBC data. In the case of resurfaced LABnb, a positively charged scaffold may provide a means for lipid A-specific CDRs to access and bind their target, thereby further destabilizing the outer membrane and killing the cell.

Protein–lipid A interactions are not uncommon, but they are usually driven by electrostatic interactions (29). The lack of charged residues in the LABnb CDRs suggests an alternative mode of binding. We demonstrate that resurfacing of LABnb was required for activity in common growth medium. Although our data hint to an alternative antibacterial mechanism from the electrostatic-based interactions of other lipid A–binding antimicrobials, we note that the introduction of cationic charges provides additional routes for LABnb to interact with lipid A. This may simply result from disruption of the outer membrane, which facilitates LABnb interactions with lipid A, but may also lead to altered CDR interactions as well. The contributions of charge and CDR interactions to the overall activity of LABnb will be pursued in future studies.

Our work introduces an exciting proof of concept for identifying antimicrobial nanobody leads from synthetic libraries and arming them with the capacity to access previously sequestered membrane targets. Our lipid A–binding nanobody would benefit from optimization to improve its activity, including evaluations of variants for enhanced lipid A interactions and overall protein stability. Most of the nanobodies investigated in this study were tolerant of cationic resurfacing, yet resurfaced LABnb exhibited significant drops in purification yield (~fourfold lower than its nonresurfaced parent). Combined with a relatively high µg/mL inhibitory concentration, this limited our ability to identify the activity range of resurfaced LABnb against bacteria. Our initial investigations of the resurfaced LABnb activity spectrum revealed bactericidal activity against *A. baumannii* and bacteriostatic activity against *P. aeruginosa.* Since we do not know how LABnb engages with lipid A, the reasons for the observed differences in activity are unclear. Previous investigations of outer membrane compositions (33–35) imply that the observed difference in sensitivity is likely not the result of variability in lipid A structure and may instead be attributed to other resistance mechanisms in *P. aeruginosa*, such as protease production (50). Both resurfaced LABnb and its scrambled variant exhibited static activity against *Staphylococcus*, indicating that these effects are likely driven by nonspecific electrostatic interactions with the gram-positive cell surface (51). Further optimization of these constructs may enhance strain specificity and improve nanobody potency.

Previous studies indicate that resurfaced nanobodies could penetrate mammalian cells under specific conditions (17). However, inherent differences in the electrostatic profiles of mammalian and gram-negative cells should allow resurfaced nanobodies to preferentially target bacterial cells with minimal collateral damage. It has been shown that cationic molecules interact differently with anionic gram-negative membranes than zwitterionic mammalian membranes (52–55). Although our red blood cell assay shows that nanobodies do not elicit hemolytic activity, further examination of the effects of resurfaced nanobodies on mammalian cells is needed to support their potential as therapeutics. Furthermore, cationic antibacterials often show toxicity from systemic administration. Thus, development of resurfaced nanobodies will have to contend with this general challenge or be positioned for nonsystemic use. Modulation of amounts and distribution of charge could help enhance cell target specificity, as has been observed for cationic peptides (56). While we only explored one nanobody in this work, we anticipate that more nanobodies targeting additional features, such as essential components of the Bam and LptD systems, could be identified through our approach.

## Methods

### Flow Cytometry.

*E. coli* W3110 containing the pMMB67EH plasmid encoding the SLAY display system (20) with anti-GFP nanobody or a tandem influenza hemagglutinin peptide (2×HA) were grown overnight in lysogeny broth (LB) supplemented with carbenicillin (75 µg/mL) at 37 °C with 220 rpm shaking. The following day, two cultures per construct were prepared by diluting overnight cultures to OD_600_ 0.1. Cells were grown at 37 °C until the exponential phase; then, one culture replicate was induced with IPTG to a final concentration of 0.1 mM. All cultures were grown for an additional 3 h at 30 °C. Cells were pelleted and resuspended to OD_600_ 0.1 in 1 mL of 1× PBS with 50 mM glucose. Recombinant GFP (Abcam ab84191) was added to cells to a final concentration of 0.2 µM, and cells were incubated at 30 °C with shaking for 30 min. Cells were gently pelleted and resuspended in 1 mL PBS-glucose. Flow cytometry measurements were taken using a Sony SA3800 spectral cell analyzer until 10,000 measurements were captured. Flow cytometry data were analyzed using FlowJo software (v. 10.10.0).

### SLAY Screen.

Our nanobody surface display screen was adapted from previous work (20). The nanobody library was generated as we previously described (25). Briefly, the display system with random nanobody variants was cloned into plasmid pMMB67EH. Plasmids were transformed into *E. coli* W3110 and colonies were pooled and stored as glycerol stocks at −80 °C until use. On the day of the screen, a library aliquot was thawed and 10 mL LB media with carbenicillin (75 µg/mL) was inoculated with the library to OD_600_ of 0.1 and grown at 37 °C with shaking for 1 h. After 1 h, cultures were diluted to OD_600_ 0.01 in 5 mL LB with carbenicillin, in duplicate for uninduced and induced conditions. To induce cultures, IPTG was added to a final concentration of 0.1 mM. All cultures were then grown at 37 °C with shaking for 3 h, after which plasmids were harvested using the Zyppy Plasmid Miniprep kit (Zymo Research Corp.). Adapters for sequencing were added using primers, and nanobody variants were sequenced using Illumina sequencing techniques. CDR sequences were compared between induced and uninduced conditions using bioinformatics approaches previously outlined (20) to identify depleted sequences. Sequencing data were uploaded to NCBI (Accession number: PRJNA908087).

### SLAY Growth Curves.

Plasmids containing the SLAY display system were transformed into *E. coli* W3110. Single colonies were inoculated into 5 mL LB media containing carbenicillin (75 µg/mL) and grown at 37 °C with 220 rpm shaking until the exponential phase. Cultures were diluted to OD_600_ 0.01 and 200 µL of diluted culture was added to each well of a 96-well plate. Appropriate wells were induced with IPTG to 0.1 mM or 1 mM final concentrations. Cultures were grown at 37 °C with shaking, and density measurements were recorded every 10 min using a BioTek Logphase 600 plate reader.

### Nanobody Expression.

Nanobodies purified in this study were cloned into pET28a between NcoI and XhoI restriction sites using standard cloning protocols. Final constructs contained a C-terminal 6x-histidine tag to enable immobilized metal affinity chromatography (IMAC). Plasmids were transformed into SHuffle T7 Express competent cells (New England Biolabs, cat #C3029J) and grown overnight at 30 °C. Colonies were then used to inoculate 25 mL LB containing 25 µg/mL kanamycin and grown overnight. All SHuffle T7 cultures used in this study were grown in LB containing kanamycin (25 µg/mL) at 30 °C with 220 rpm shaking. The following day, overnight cultures were equally divided and used to inoculate two 425 mL of LB. Cultures were grown to OD_600_ of 0.5, induced with IPTG to a final concentration of 1 mM, and grown for an additional 5 h. Cells were then pelleted using a Beckman Coulter centrifuge (JA-10 fixed angle rotor) at maximum speed (10,000 rpm) for 10 min. Two rounds of centrifugation were run to collect cells from both cultures for each construct. Bacterial pellets were stored at −80 °C until proceeding with purification. All nanobodies were purified within 1 wk of freezing.

### Nanobody Purification.

#### IMAC.

All buffers used for purification were filter-sterilized (0.22 µm) and stored at 4 °C. Frozen bacterial pellets were resuspended in lysis buffer (50 mM phosphate, 1 M NaCl, 10 mM imidazole, pH 7.4) containing protease inhibitor (Pierce cat# A32963) dissolved according to the manufacturer’s instructions. Lysis buffer was added to a final cell suspension volume of 30 mL. Cells were then lysed via two consecutive passes through a chilled French press. (Glen Mills, 40 K Cell FA-032) at ~16,000 psi. Lysates were centrifuged (Beckman Coulter, JA-17 fixed angle rotor) at 17,000 rpm for 15 min. The centrifugation step was repeated, and supernatants were transferred to chilled 50 mL conical tubes. IMAC purification steps were conducted at 4 °C. HisPur Cobalt Resin (Thermo Scientific, cat. # 89965) was gently resuspended in storage buffer and 500 µL of cobalt resin slurry was loaded into a disposable chromatography column (Bio-Rad, cat. #7311550) and the resin bed was equilibrated with lysis buffer. Lysate was added onto the equilibrated resin bed and allowed to flow through the column via gravity flow. Once all of the lysate passed through the column, the resin bed was washed with 30 mL of lysis buffer. Proteins were eluted from the cobalt resin in 250 µL fractions using elution buffer (50 mM phosphate, 1 M NaCl, 150 mM imidazole, pH 7.4). Protein concentrations were preliminarily measured using absorbance reads at A280nm (NanoDrop 2000 Spectrophotometer), and fractions were combined based on concentration. For GFPnb, Ng2nb, and their resurfaced counterparts, fractions were combined and purified in a second step using size exclusion chromatography (SEC). For LABnb, scrambled LABnb, and their resurfaced variants, eluted fractions were dialyzed after IMAC.

#### SEC.

After IMAC purification, nanobodies were purified using a Superdex 200 Increase 10/300 GL column (Cytiva 28990944) mounted on ÄKTA Pure 25 Chromatography System. The Superdex column was equilibrated with 1 column volume of phosphate buffer (50 mM phosphate, 1 M NaCl, pH 7.4) prior to loading 1 mL of IMAC-purified nanobody sample. Proteins were eluted from the column in 200 µL fractions. Fractions that eluted at the expected nanobody size were collected and combined for dialysis.

#### Dialysis.

After purification, samples were loaded into SnakeSkin dialysis tubing (7 kDa MW cut-off; Thermo Scientific, cat. #68700). Sealed dialysis bags were incubated at 4 °C overnight in dialysis buffer (50 mM phosphate, 150 mM NaCl, pH 7.4) at a ratio of 1:500 (sample: dialysis buffer). After overnight incubation, dialysis buffer was replaced with fresh buffer, and samples were incubated for an additional 4 h.

#### Nanobody collection, concentration measurements, and storage.

After the final dialysis incubation, nanobody samples were transferred to Protein Lo-Bind microcentrifuge tubes (Eppendorf, cat. # 022431102) and centrifuged (14 k *g*, 10 min, 4 °C) to collect any precipitate. Supernatants were transferred to fresh microcentrifuge tubes, and protein concentrations were measured using the Pierce BCA Protein Assay kit (cat. #23225), according to the manufacturer’s protocol. BCA-quantified concentrations were used to determine nanobody concentrations for all downstream assays. Proteins were aliquoted, snap frozen in liquid nitrogen, and stored at −80 °C until use.

### Protein Gel and Immunoblotting.

Nanobodies were diluted to equal micromolar concentrations in filtered dialysis buffer, mixed with SDS buffer, and incubated at 99 °C for 10 min. Samples were loaded onto NuPAGE 4-12% Bis-Tris protein gels (Invitrogen, cat. #NP0321BOX) and run in MES buffer. For Coomassie-staining, gels were incubated with SimplyBlue SafeStain (Invitrogen, cat. #LC6065). For immunoblotting, proteins were transferred onto PVDF transfer membranes (0.45 µm; Thermo Scientific cat. #88518). After transfer, membranes were incubated in blocking buffer (1x Tris-buffered saline, 0.1% Tween-20, 5% powdered milk) for 1 h. Nanobodies were detected using anti-nanobody (VHH) antibodies conjugated with horseradish peroxidase (HRP; GenScript, cat. # A01861) diluted in blocking buffer (1:5000 antibody to milk ratio). Membranes were incubated with antibodies overnight at 4 °C with rocking. The following day, membranes were washed with three 5 min washes in blocking buffer, followed by three 5 min washes in TBST (1xTris-buffered saline, 0.1% Tween-20). Membranes were incubated with 1 mL substrate (ThermoFisher Scientific, cat. #34095) and imaged with a Bio-Rad ChemiDoc Imaging System.

### Immunoprecipitation.

For each nanobody tested, 50 µL of Dynabeads His-Tag Isolation and Pulldown beads (Invitrogen, cat #10103D) were washed twice in lysis buffer (50 mM phosphate, 1 M NaCl, 10 mM imidazole, pH 7.4) and resuspended in 1 mL of lysis buffer. To the beads, 100 µg of purified nanobodies (either anti-GFPnb or LABnb) were added, and samples were rotated overnight at 4 °C. Beads were then washed three times with lysis buffer to remove unbound protein. *E. coli* W3110 lysate was prepared from a 200 mL culture grown at 37 °C. Cells were pelleted, resuspended in lysis buffer, and sonicated. Lysate was pelleted twice (14k rpm, 5 min), and 1 mL of clarified supernatant was added to the washed beads. Samples were rotated for 90 min at 4 °C, then washed with 10 mL of lysis buffer to remove nonspecific interactions. Beads were boiled in 50 µL SDS buffer, and 20 µL of samples was run on a PAGE gel. Proteins were visualized using Coomassie stain.

### Circular Dichroism.

Purified nanobodies were diluted in 10 mM potassium phosphate buffer to 50 µM in 200 µL. Samples were measured using a Jasco-815 CD spectrometer using a 190 to 260 nm measurement range. All data were baseline corrected, and measurements represent averages of three accumulations collected from the same sample. Reported mdeg data from the experiment were used to calculate molar ellipticity values.

### Lipid A ELISA.

Lipid A diphosphoryl (Sigma-Aldrich, Product no. L5399) was diluted to 5 µg/mL in chloroform:methanol (1:9), and 50 µL of the solution was loaded into each well of a 96-well plate (Costar 96-well EIA/RIA plate, ref#3590). The plate was sealed with a foil adhesive and was incubated overnight at 4 °C with gentle rocking. The following day, wells were emptied and washed once with 100 µL of 1× PBS. Each well was then loaded with 300 µL of PBS + 0.5% BSA, sealed with adhesive foil, and incubated at 37 °C for 3 h. Wells were emptied and washed with 300 µL of 1× PBS. Purified nanobody samples were normalized to the same starting concentrations in filtered dialysis buffer and then diluted to 10 µg/mL and 5 µg/mL concentrations in 1× PBS + 0.5% BSA. Appropriate wells were then loaded with 50 µL of nanobody samples, sealed with adhesive foil, and incubated at 4 °C overnight with rocking. The following day, liquid was dumped from the plate, wells were washed five times with 200 µL of 1× PBS, and wells were incubated with 50 µL of PBS + 0.5% BSA containing anti-VHH antibodies conjugated with HRP (diluted 1:5000). The plate was sealed and incubated at room temperature in the dark with rocking for 1 h. Wells were washed five more times with 200 µL 1× PBS, and each well was loaded with 50 µL 1-Step Ultra TMB-ELISA substrate (Thermo Scientific, cat. #34028). After developing, wells were immediately quenched with 50 µL of 2 M H_2_SO_4_, and absorbance (450 nm) was measured on a BioTek Synergy LX plate reader.

### MIC and MBC Assays.

*E. coli* W3110 was inoculated into 5 mL Mueller Hinton broth (MHB) and grown at 37 °C with 220 rpm shaking to OD_600_ of 0.5. For each nanobody, 100 µL of nanobody diluted to 128 µM in dialysis buffer was added to a 96-well polypropylene plate (Corning, ref. #3879), in triplicate. Nanobodies were serially diluted two-fold in dialysis buffer within the plate. Bacterial cultures were diluted in MHB to OD_600_ of 0.001 and 50µL of diluted cells were added to each well. Plates were sealed with parafilm and incubated at 37 °C for 18 h. MIC values were determined according to criteria established by EUCAST. For MBC measurements, 5 µL from each well was spotted onto LB-agar and incubated at 37 °C overnight. Plates were inspected the following day, and clearance was used to infer MBC cutoffs.

### Tris MBC.

*E. coli* W3110 cultures were grown as outlined above. After reaching OD_600_ of 0.5, 500 µL of culture was pelleted (4k rpm, 10 min), washed with 1 mL Tris buffer (50 mM Tris, 25 mM NaCl, 0.05% glucose, pH 7.4), and resuspended in Tris buffer to a final density of OD_600_ 0.001. Nanobody concentrations were normalized in dialysis buffer, then diluted in Tris buffer as described above for the MIC assay. Cells resuspended in Tris buffer were added to the plate, and MBC determinations were carried out as described above.

### 1-*N*-Phenylnaphthylamine Assay.

The 1-*N*-phenylnaphthylamine (NPN) assay used in this study was based on methods from a previous publication (32). *E. coli* W3110 colonies were used to inoculate 25 mL LB media, and the culture was grown at 37 °C with 220 rpm shaking until OD_600_ 0.5. Cells were pelleted (4k rpm, 10 min), washed with HEPES-glucose buffer (5 mM HEPES, 5 mM glucose, pH 7.4), and resuspended in HEPES-glucose buffer to a final OD_600_ of 0.5. A working solution of 40 µM NPN was prepared by diluting a 0.5 mM NPN stock solution (dissolved in acetone) with HEPES-glucose buffer. Purified nanobodies were normalized to the same concentration in dialysis buffer. Each experimental well of a 96-well plate was loaded with 50 µL diluted nanobodies (to a final concentration of 10 µM), 50 µL NPN working solution, and, added last, 100 µL bacterial cell suspension. The colistin positive control was prepared by diluting a 2 mg/mL stock in dialysis buffer. Each sample was tested in triplicate. Fluorescence was measured over the course of 1 h using a BioTek Synergy LX plate reader (excitation 360/40, emission 460/40).

### Propidium Iodide Assay.

*E. coli* W3110 cultures were grown as described for the NPN assay. After reaching OD_600_ of 0.5, cells were pelleted (4k rpm, 10 min) and washed twice with 1× PBS. Cells were resuspended in 1× PBS with 50 mM glucose to a final density of OD_600_ 0.1. Propidium iodide solution was added to the cell suspension to a final concentration of 10 µg/mL. Nanobodies and control treatments (colistin and carbenicillin) were diluted in dialysis buffer to normalized concentrations, and 50 µL of the diluted samples was added in triplicate to the wells of a black optical bottom 96-well plate (Thermo Scientific, cat #265301). Next, 50 µL of the cells-propidium iodide mixture was added to each well and the plate was incubated in the dark at 37 °C for 25 min. After incubation, the plate was equilibrated to room temperature, and measurements were collected using a BioTek Synergy LX plate reader (excitation 530/25, emission 590/35).

### Fractionation.

*E. coli* cultures were grown as detailed in the NPN assay section. After reaching the appropriate density, 4 mL of culture was transferred to a 15 mL conical tube, and 1 mL of purified nanobodies diluted in dialysis buffer was added to the culture (final nanobody concentration = 10 µM). Nanobodies were incubated with cells at room temperature for 30 min. After incubation, cells were centrifuged (4k rpm, 10 min) and cell pellets were used for either total membrane of periplasm fractionation.

#### Total membrane fractions.

The cell pellet was resuspended in 1 mL buffer 1 (10 mM Tris and 150 mM NaCl, pH 7.3), and the cell suspension was lysed using one pass through a French press. miniature pressure cell (~15,000 psi). Cell lysates were pelleted at 12k *g* for 30 min at 4 °C. Supernatants were transferred to tubes (Beckman Coulter, ref #349623) and centrifuged at 50 k rpm for 45 min at 4 °C. The remaining supernatant was removed with a pipette, and the pellet was resuspended in 200 µL buffer 2 (50 mM Tris and 150 mM NaCl, pH 7.3).

#### Periplasm fractions.

The cell pellet was washed with 500 µL of cold buffer 1 and resuspended in 300 µL SET buffer (0.5 M sucrose, 1 mM EDTA, and 200 mM Tris-HCl, pH 7.3). Lysozyme solution (0.5 mg dissolved in 50 µL water) was then added to the cell suspension, followed by 300 µL ice-cold water. The sample was incubated at 37 °C for 30 min, then pelleted (12k *g*, 30 min, 4 °C). Supernatant (periplasm fraction) was transferred to a microcentrifuge tube.

#### Fractionation immunoblots.

Protein gels and immunoblots were run as described above. After the initial 1 h incubation in blocking buffer, membranes were cut at the appropriate kDa marker positions to allow for incubation with different antibodies. For nanobody detection, anti-VHH antibodies were used, as previously described. Primary control antibodies (anti-MBP and anti-OmpA) were diluted in blocking buffer (1:5000 antibody:milk) and membranes were blocked overnight. After three 5-min washes in blocking buffer, membranes were incubated with secondary HRP-conjugated antibodies diluted 1:1500 in blocking buffer for 4 h at 4 °C. Final washing steps and imaging were carried out as outlined above, and each membrane panel was imaged independently.

### SEM.

An overnight culture of *E. coli* W3110 was grown in LB broth for 18 h. The cells were centrifuged, washed two times in MHB, and subcultured (1:100) in MHB supplemented with 0.223 mM CaCl_2_. The cells were grown to OD_600_ 0.8 and then standardized to a final OD_600_ 1.2 using double strength MHB. The cells were then mixed 1:1 with buffer (50 mM phosphate, 150 mM NaCl, pH 7.4), 80 μM resurfaced GFPnb, 80 μM resurfaced scrambled LABnb, 80 μM LABnb, 80 μM resurfaced LABnb, or 4 μg/mL of colistin; this yielded a final OD_600_ at 0.6 and an effective concentration of 40 μM for all the nanobodies and 2 μg/mL for colistin. After a 2.5-h incubation period at 37 °C, 25 μL of each sample was spotted onto a positively charged glass microscope slides and allowed to air-dry. Cells were fixed with glutaraldehyde (2.5% v/v in 1x PBS) for 30 min at room temperature, followed by washing five times with 1× PBS. Each sample was then dehydrated using an increasing concentration of ethanol [5, 10, 20, 30, 50, 70% v/v (applied three times), 90% v/v (applied three times) and 100% v/v]; each wash was carried out by application and immediate removal of the alcohol solution. A 5-nm coat of platinum/palladium was applied using a Cressington 208 benchtop sputter coater. Images were obtained on a Zeiss Gemini 460 Scanning Electron Microscope with EHT at 5.00 kV and iProbe at 200 pA.

### Hemolysis Assay.

Human red blood cells (Innovative Research, IWB3ALS) were washed twice in dialysis buffer and resuspended to a cell density of 8 × 10^8^ cells/mL in dialysis buffer. Purified nanobodies and Triton X-100 were diluted in dialysis buffer to twice the desired final concentrations, and 100 µL of each treatment was added to the wells of a polypropylene plate. To each well, 100 µL of red blood cells were added (final cell density = 4 × 10^8^ cells/mL) and the plate was incubated at 37 °C for 3 h. After incubation, the plate was spun down (600×*g*, 5 min, 4 °C) and the supernatant was carefully pipetted off and transferred to a flat-bottom optical well plate. Absorbance was measured at 540 nm, and hemolysis was calculated relative to the average Triton X-100 reads (i.e., 100% hemolysis).

## Supplementary Material

Appendix 01 (PDF)

Dataset S01 (XLSX)

## Data Availability

Sequencing data have been deposited in NCBI Sequence Read Archive (PRJNA908087) (57).
