# Supplementary material for: Resurfacing promotes antibacterial activity of a lipid A–binding nanobody
Source: Proc Natl Acad Sci U S A. 2025 Sep 3;122(36):e2509305122. doi: 10.1073/pnas.2509305122 (PMC12435215; doi:10.1073/pnas.2509305122)
Supplement: Supplementary file 1 — Appendix 01 (PDF) [file pnas.2509305122.sapp.pdf]

Supporting Information for

Resurfacing promotes antibacterial activity of a lipid A-binding nanobody

Angela C. O'Donnell[a], Xun Wang[a,1], Nikol Kadeřábková[a], Kyra E. Groover[a], Bethany C. Perez[a], Amanda Helms[b], Jennifer S. Brodbelt[b], Despoina A. I. Mavridou[a,c], Bryan W. Davies[a,c,2]

[a]Department of Molecular Biosciences, The University of Texas at Austin, Austin, TX 78712

[b] Department of Chemistry, The University of Texas at Austin, Austin, TX, 78712

[c]John Ring LaMontagne Center for Infectious Diseases, The University of Texas at Austin, Austin, TX 78712

[2]Corresponding Authors: Bryan W. Davies, bwdavies@utexas.edu

[1]Current Address: Lawrence Livermore National Laboratory

This PDF file includes:

Figures S1 to S14

Other supporting materials for this manuscript include the following:

Dataset S1

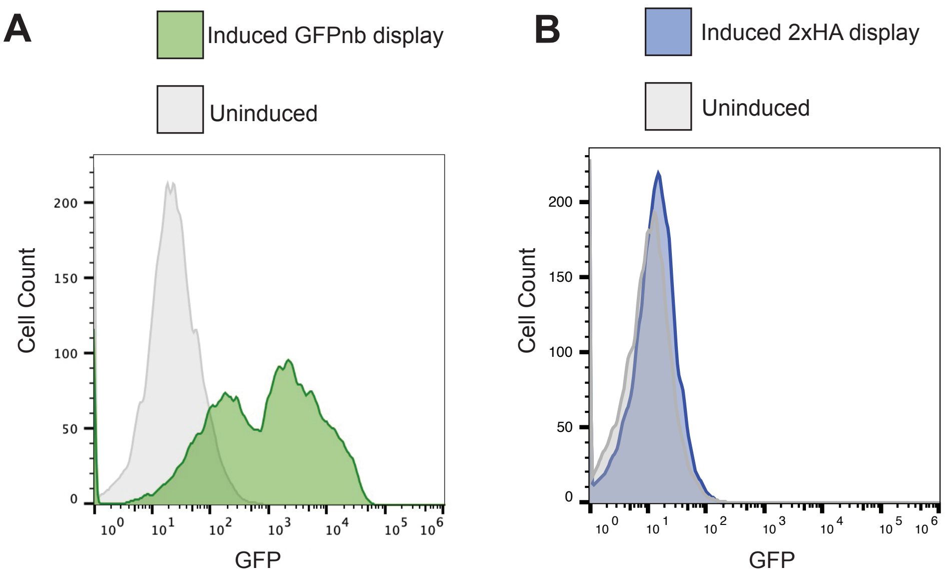

Figure S1. Flow cytometry data showing measured GFP fluorescence. **A.** *E. coli* cultures induced to express anti-GFP nanobody using our surface display system are represented by the green peak and the corresponding uninduced *E. coli* data appear in grey. The shift to the right (green peak) indicates that the display of anti-GFP nanobodies results in GFP fluorescence, suggesting binding. **B.** No shift in GFP fluorescence is detected in *E. coli* containing the display system with a tandem influenza hemagglutinin peptide (2XHA) in either the uninduced (grey peak) or induced (purple peak) populations.

A

>Library scaffold | MAEVQLQASGGGFVQAGGSLRLSCAASGXXXXXXXMGWFRQAPGKEREFVAAISXXXXXXXYYADSVKGRFTISRDNAKNTVYLQMNSLKPEDTATYYCAXXXXXXXXXYWGQGTQVTVSSLEHHHHHH

B

>LAB nanobody | MAEVQLQASGGGFVQAGGSLRLSCAASGDGYTDSIMGWFRQAPGKEREFVAAISGEDSEKQYYADSVKGRFTISRDNAKNTVYLQMNSLKPEDTATYYCASEIIYSIQQYWGQGTQVTVSSLEHHHHHH

Complementarity determining regions

Figure S2. A. Sequence of nanobody scaffold used for display library. Library sequence variation was introduced at complementarity determining regions (CDRs), highlighted yellow. **B.** Sequence of LABnb nanobody demonstrating lipid A-binding CDRs obtained from antimicrobial screen.

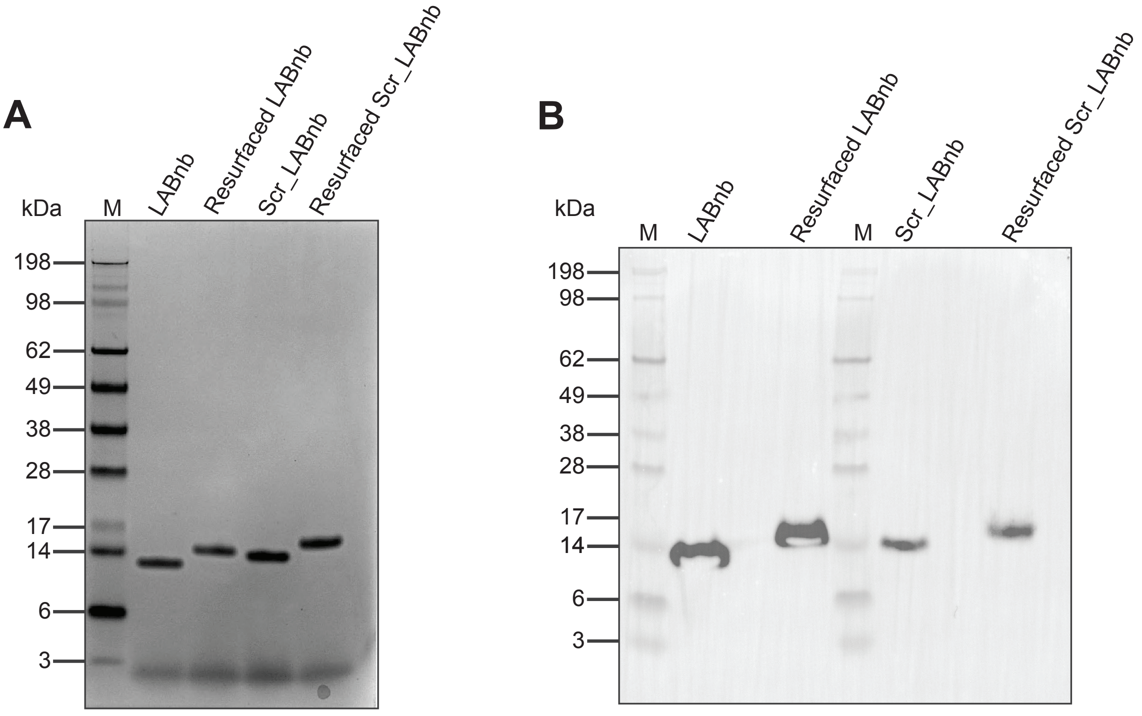

Figure S3. A. Coomassie-stained protein gel showing purified nanobodies loaded at equal molar concentrations. **B.** Immunoblot displaying purified nanobodies detected with anti-VHH antibodies conjugated with horseradish peroxidase (HRP). Equal molar concentrations of purified nanobodies were loaded into the gel. Approximate molecular weights (in kDa) are denoted by protein size marker (M) columns.

A

```
>LABnb                      MAEVQLQASGGGFVQAGGSLRLSCAASGDGYTDSIMGWFRQAPGKEREFVAAISGEDSEKQYYADSVKGRFTISRDNAKNTVYLQMNSLKPEDTATYYCASEIIYSIQQYWGQGTQVTVSSLEHHHHHH
>Resurfaced LABnb           MAEVQLQAKGGKRVQAGGSLRLKCAASGDGYTDSIMGWFRQAPGKEREFVAAISGEDSEKQYYADSVKGRFKIKRDNAKNTVYLRMRKLKPEDTATYYCASEIIYSIQQYWGQGTRVTVSSLEHHHHHH

>Scrambled LABnb            MAEVQLQASGGGFVQAGGSLRLSCAASGSDIEGQYMGWFRQAPGKEREFVAAISIGSDQTYYYADSVKGRFTISRDNAKNTVYLQMNSLKPEDTATYYCADEISQSEKIYWGQGTQVTVSSLEHHHHHH
>Scrambled resurfaced LABnb MAEVQLQAKGGKRVQAGGSLRLKCAASGSDIEGQYMGWFRQAPGKEREFVAAISIGSDQTYYYADSVKGRFKIKRDNAKNTVYLRMRKLKPEDTATYYCADEISQSEKIYWGQGTRVTVSSLEHHHHHH
```

B

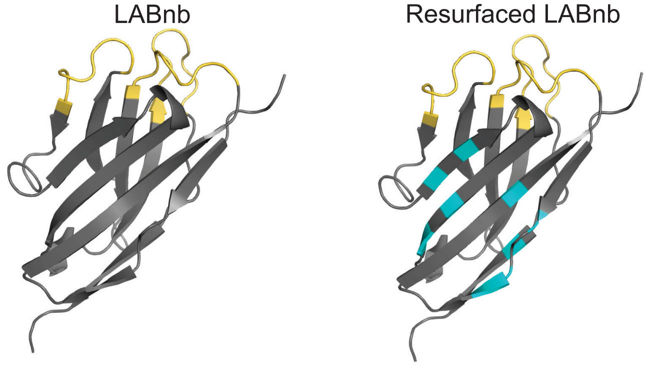

Figure S4. A. Amino acid sequences of nanobody LABnb and its scrambled and resurfaced variants. Sequences of lipid A binding CDRs are highlighted yellow and scrambled CDRs are highlighted grey. Residues modified for scaffold cationic resurfacing are highlighted in cyan. **B.** AlphaFold2[1] predicted structures of nanobody LABnb and resurfaced nanobody LABnb. CDRs appear in yellow and resurfaced residues are shown in cyan.

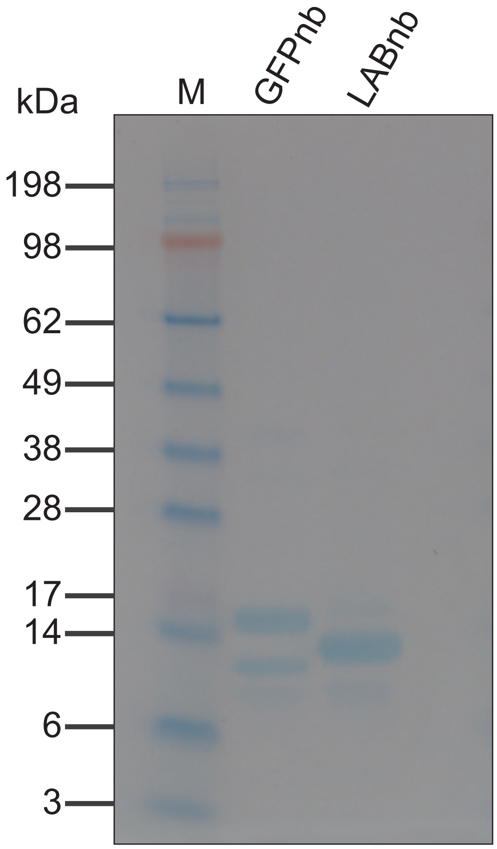

Figure S5. Coomassie-strained protein gel showing immunoprecipitation samples of *E. coli* lysate incubated with either GFPnb or LABnb bound to superparamagnetic beads. Protein size marker (M) shows approximate molecular weights in kDa. Expected size of protein bands for nanobodies is ~14 kDa.

A

>GFPnb MAQVQLVESGGALVQPGGSLRLSCAASGFPVNRYSMRWYRQAPGKEREWVAGMSSAGDRSSYEDSVKGRFTISRDDARNTVYLQMNSLKPEDTAVYYCNVNVGFEYWGQGTQVTVSSKLERPHRDLEHHHHHH

>Resurfaced GFPnb MAQVQLVEKGGKRVQPGGSLRLKCAASGFPVNRYSMRWYRQAPGKEREWVAGMSSAGDRSSYEDSVKGRFKIKRDDARNTVYLRMRKLKPEDTAVYYCNVNVGFEYWGQGTRVTVSKKLERPHRDLEHHHHHH

>Ng2nb MAEVQLQASGGGFVQAGGSLRLSCAASGGTSDATTMGWFRQAPGKEREFVAAISSYNTMLFYYADSVKGRFTISRDNAKNTVYLQMNSLKPEDTATYYCATSSTVNHWDYWGQGTQVTVSSLEHHHHHH

>Resurfaced Ng2nb MAEVQLQAKGGKRVQAGGSLRLKCAASGGTSDATTMGWFRQAPGKEREFVAAISSYNTMLFYYADSVKGRFKIKRDNAKNTVYLRMRKLKPEDTATYYCATSSTVNHWDYWGQGTRVTVSSLEHHHHHH

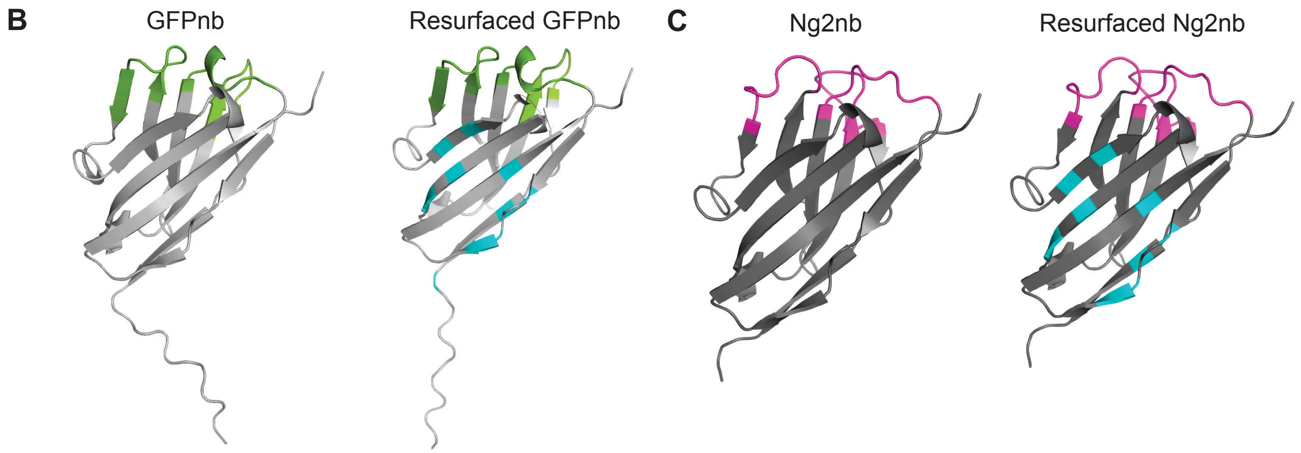

Figure S6 A. Amino acid sequences of anti-GFP nanobody, nanobody Ng2, and their resurfaced counterparts. CDR sequences are highlighted green (for GFPnb) or magenta (for Ng2nb). Residues highlighted in cyan were selected for cationic resurfacing. **B.** AlphaFold2[1] predicted structures of anti-GFP nanobody and resurfaced anti-GFP nanobody. Scaffold regions are shown in light grey, CDRs appear in green, and resurfaced residues are shown in cyan. **C.** AlphaFold2[1] predicted structures of Ng2 nanobody and resurfaced Ng2 nanobody. Scaffold regions are shaded dark grey. CDRs are shown in magenta, and resurfaced residues are highlighted cyan.

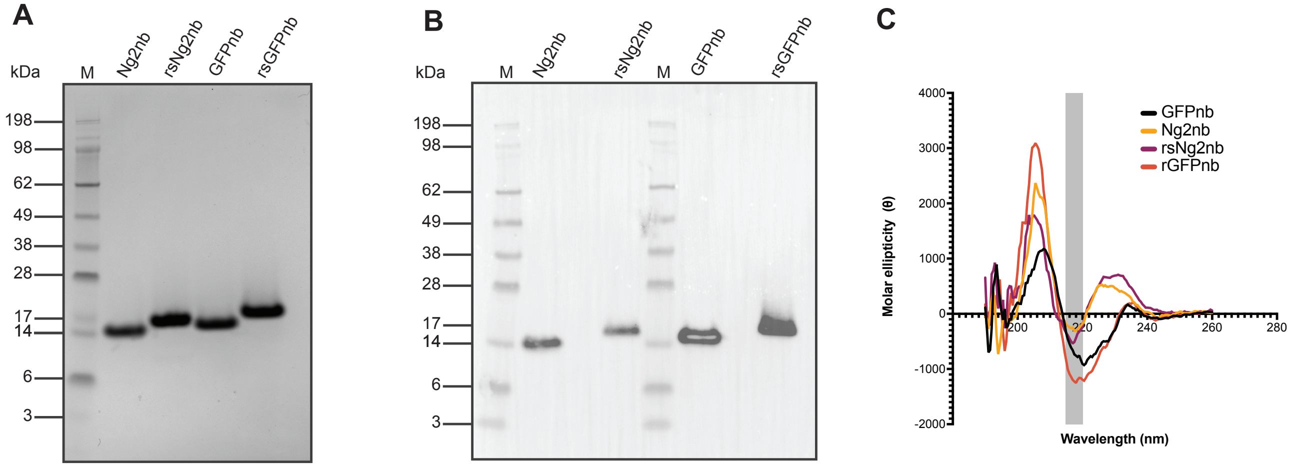

Figure S7. A. Coomassie-stained protein gel showing equal molar concentrations of purified nanobodies. **B.** Immunoblot displaying purified nanobodies detected with anti-VHH antibodies conjugated with HRP. Equal molar concentrations of proteins were loaded into the gel. Estimated molecular weights (in kDa) are indicated by protein size marker columns (M). **C.** Circular dichroism spectra for purified nanobodies represented by molar ellipticity (θ). Grey shaded area denotes the minimum range expected for beta sheet structure.

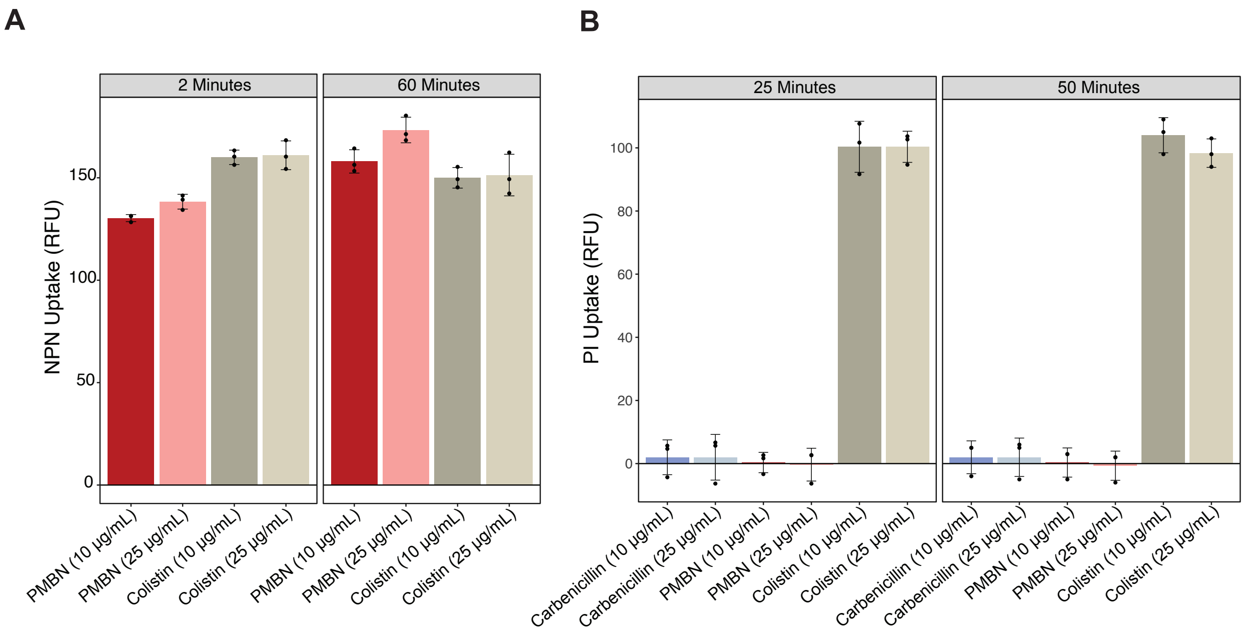

Figure S8. A. NPN relative fluorescence (RFU) of *E. coli* W3110 cells treated with polymyxin B nonapeptide (PMBN) or colistin. Average background fluorescence of treatments with only buffer were subtracted for each timepoint. **B.** Propidium iodide relative fluorescence (RFU) of *E. coli* W3110 treated with carbenicillin, PMBN, or colistin. Background fluorescence measurements were subtracted from all reads. Error bars represent standard deviation, and points represent individual reads from an experiment conducted in triplicate.

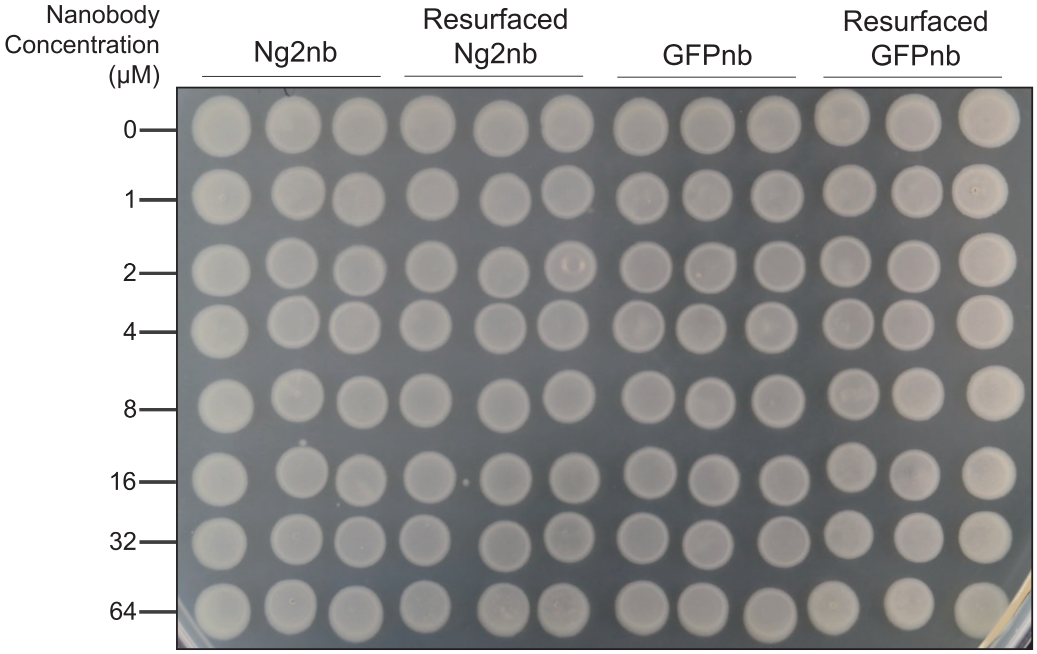

Figure S9. MBC data representing *E. coli* W3110 treated with purified nanobodies. No bactericidal activity is observed for any of the tested nanobodies at the tested concentrations (up to 64 μM). Nanobody concentrations are shown in μM. Maximum tested concentrations of 64 μM correspond to 907 μg/mL for Ng2nb, 931 μg/mL for resurfaced Ng2nb, 961 μg/mL for GFPnb, and 989 μg/mL for resurfaced GFPnb.

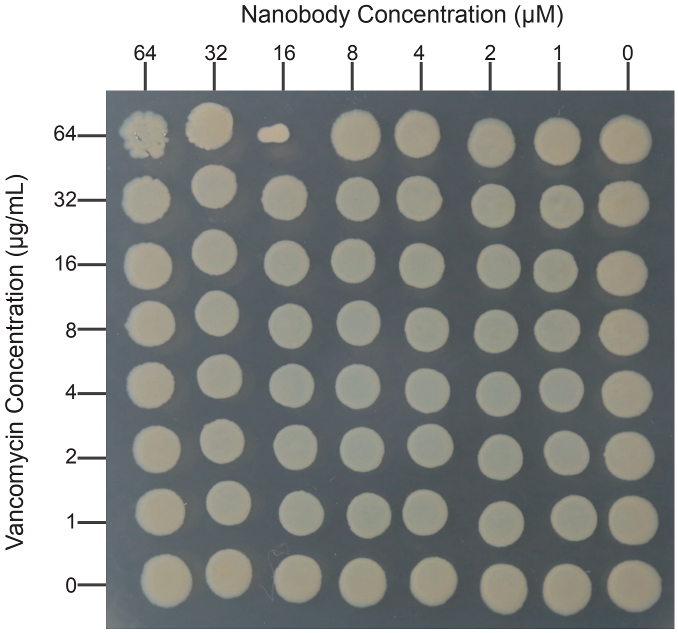

Figure S10. Bactericidal effects of resurfaced GFP nanobodies in combination with vancomycin. *E. coli* W3110 cells were treated with resurfaced GFPnb at concentrations ranging from 0 - 64μM (0 - 989 μg/mL) and vancomycin at concentrations between 0 - 64 μg/mL. Treated cultures were spotted on LB after overnight incubation at 37°C. No clearing was observed for any of the treatment combinations.

A

	Resurfaced LABnb		Resurfaced Scr_LABnb	
**Species and strain**	**Range (μM)**	**Mode (μM)**	**Range (μM)**	**Mode (μM)**
Acinetobacter baumannii **ATCC 17978**	8 to 16	16	>64	>64
Pseudomonas aeruginosa **PAO1**	32 to >64	32	>64	>64
Staphylococcus aureus **ATCC 43300**	16 to 32	16	8	8
Staphylococcus epidermidis **ATCC 12228**	16 to 32	16	8	8

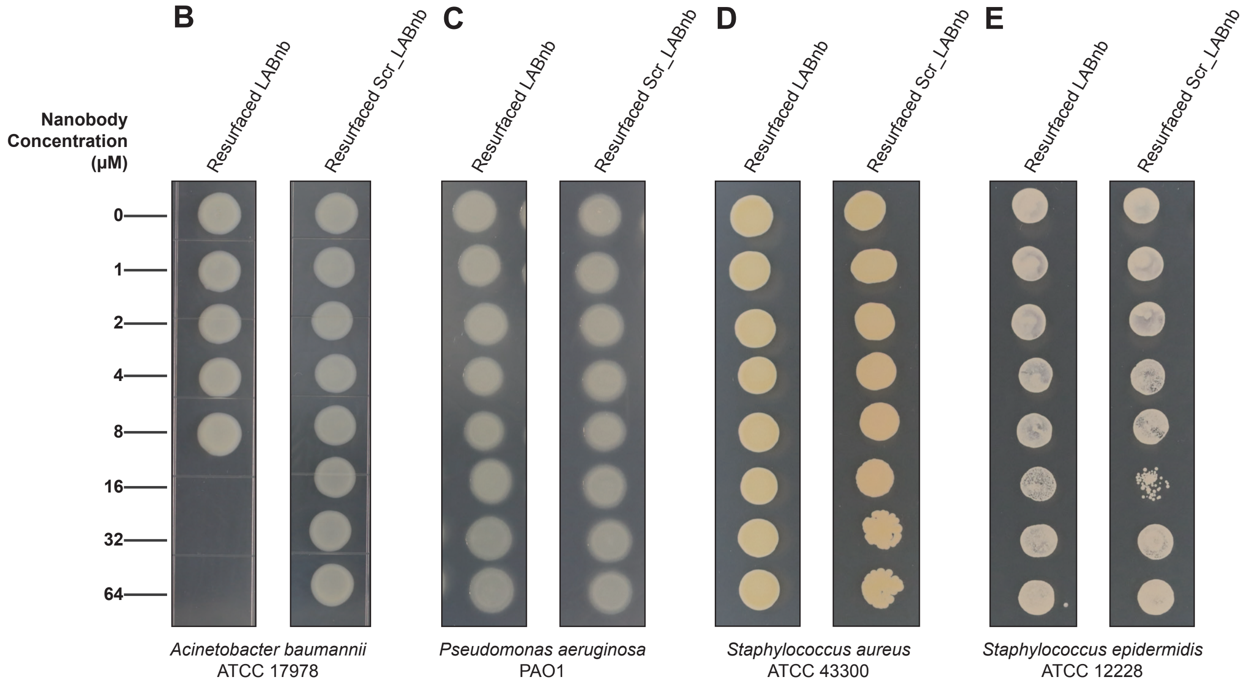

Figure S11. A. MIC results for bacterial strains treated with resurfaced LABnb and its scrambled variant. The range and most frequently observed MICs are reported in μM concentrations (64 μM = 935 μg/mL). MBC data for resurfaced LABnb and resurfaced scrambled LABnb against **B.** *Acinetobacter baumannii* (ATCC 17978), **C.** *Pseudomonas aeruginosa* (PAO1), **D.** *Staphylococcus aureus* (ATCC 43300), and **D.** *Staphylococcus epidermidis* (ATCC 12228).

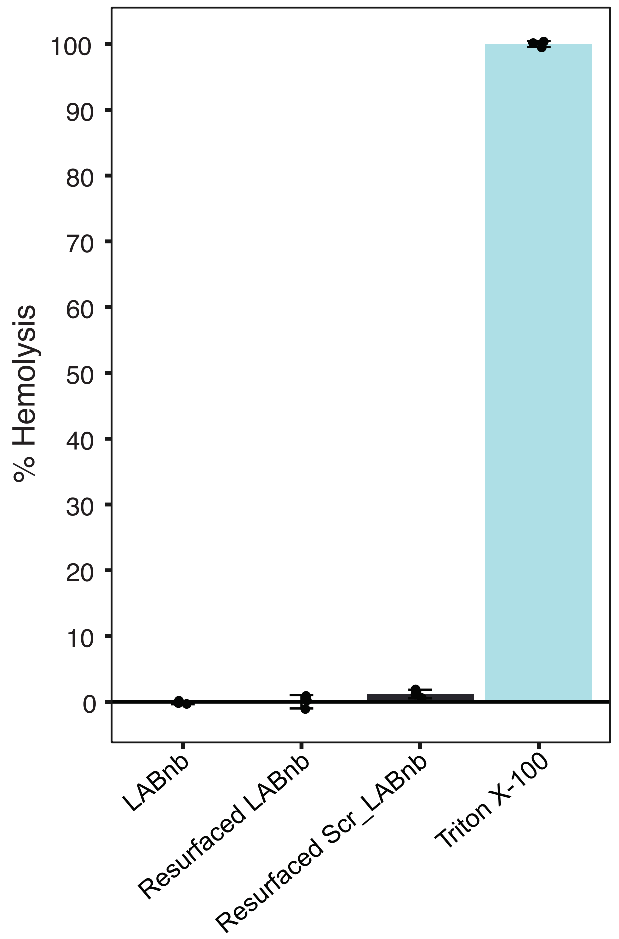

Figure S12. Hemolysis data for human red blood cells treated with nanobodies or Triton X-100. Each nanobody is shown at 64 μM concentration. Percent hemolysis was calculated relative to the 1% Triton X-100 treatment which was inferred to represent 100% expected hemolysis. Error bars denote standard deviation, and individual points represent triplicate reads within one experiment.

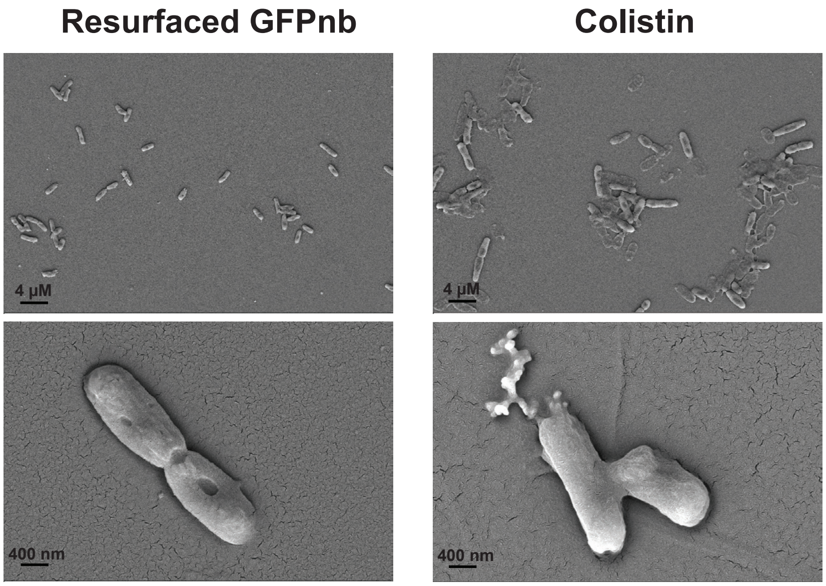

Figure S13. Scanning electron microscopy images of *E. coli* treated with resurfaced anti-GFP nanobodies (left panel) or colistin (right panel). Minimal membrane perturbation is observed from the resurfaced nanobody. Membrane disruption is visible for cells treated with colistin.

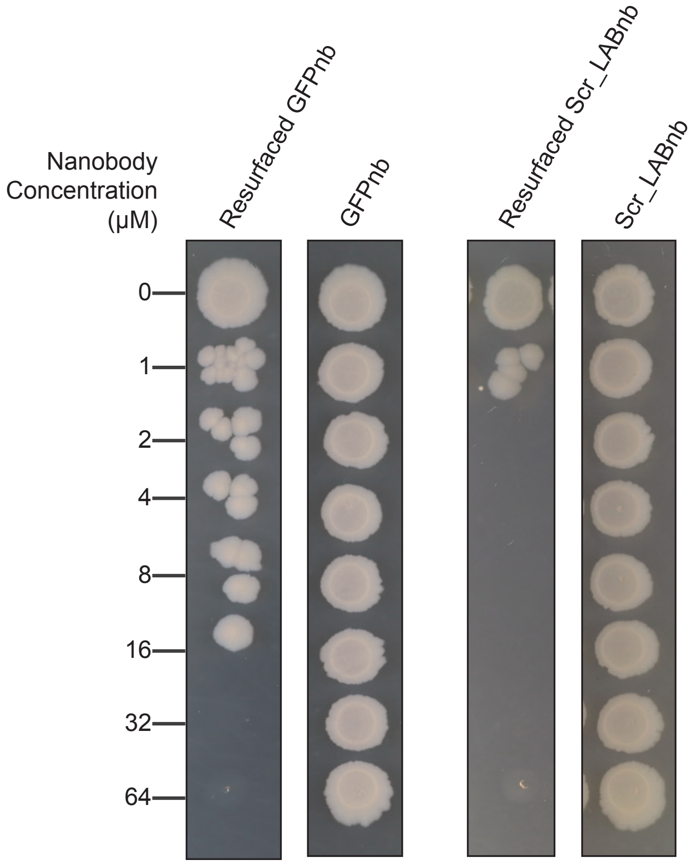

Figure S14. MBC results showing *E. coli* W3110 sensitized with Tris and treated with either resurfaced GFPnb, GFPnb, resurfaced scrambled LABnb, or scrambled LABnb. Resurfaced nanobodies demonstrate bactericidal activity when combined with the sensitizing effects of Tris buffer.

SI References

1. Jumper, J., Evans, R., Pritzel, A. *et al.* Highly accurate protein structure prediction with AlphaFold. *Nature* **596**, 583–589 (2021).
